# Application of the Adiabatic Connection Random Phase Approximation to Electron–Nucleus Hyperfine Coupling Constants

**DOI:** 10.1021/acs.jpca.4c03794

**Published:** 2024-08-20

**Authors:** Florian Bruder, Florian Weigend, Yannick J. Franzke

**Affiliations:** † Fachbereich Chemie, Philipps-Universität Marburg, Hans-Meerwein-Straße 4, 35032 Marburg, Germany; ‡ Otto Schott Institute of Materials Research, Friedrich Schiller University Jena, Löbdergraben 32, 07743 Jena, Germany

## Abstract

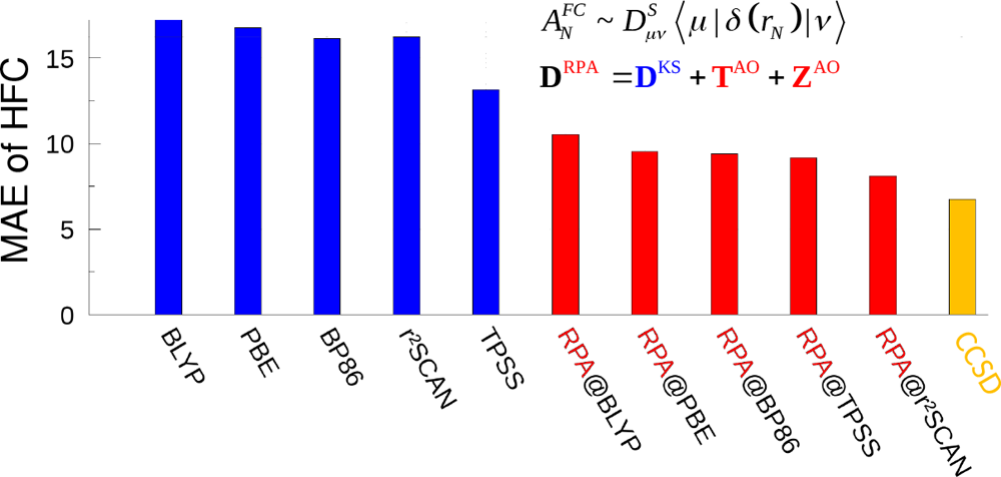

The electron–nucleus hyperfine coupling constant is a challenging property for density functional methods. For accurate results, hybrid functionals with a large amount of exact exchange are often needed and there is no clear “one-for-all” functional which describes the hyperfine coupling interaction for a large set of nuclei. To alleviate this unfavorable situation, we apply the adiabatic connection random phase approximation (RPA) in its post-Kohn–Sham fashion to this property as a first test. For simplicity, only the Fermi-contact and spin–dipole terms are calculated within the nonrelativistic and the scalar-relativistic exact two-component framework. This requires to solve a single coupled-perturbed Kohn–Sham equation to evaluate the relaxed density matrix, which comes with a modest increase in computational demands. RPA performs remarkably well and substantially improves upon its Kohn–Sham (KS) starting point while also reducing the dependence on the KS reference. For main-group systems, RPA outperforms global, range-separated, and local hybrid functionals—at similar computational costs. For transition-metal compounds and lanthanide complexes, a similar performance as for hybrid functionals is observed. In contrast, related post-Hartree–Fock methods such as Møller–Plesset perturbation theory or CC2 perform worse than semilocal density functionals.

## Introduction

Molecular systems with an open-shell configuration such as organic radicals, transition-metal catalysts, or lanthanide complexes play an important role in many fields of chemistry and materials science. These open-shell systems are routinely characterized with electron paramagnetic resonance (EPR) spectroscopy.^1−5^ Here, the g-tensor and the electron–nucleus hyperfine coupling (HFC) tensor are among the decisive quantities to interpret the respective EPR spectra. The g-tensor describes the interaction of the electron spin with the external magnetic field, whereas the HFC describes the interaction of the electron spin and a nuclear magnetic moment. This hyperfine tensor is made up of the Fermi-contact (FC), spin–dipole (SD), and the paramagnetic spin–orbit (PSO) interaction. In a simple nonrelativistic picture, the FC term is directly related to the spin-excess density at the origin of the nucleus and purely isotropic, whereas the SD term constitutes the anisotropy of the tensor.^6−10^ Inclusion of scalar-relativistic effects essentially leads to modifications of the respective FC and SD operators but the spin-density in the vicinity of the nucleus is still the key quantity.^11−18^ Overall, the FC term is of great interest for single molecule magnets (SMMs) and their application as molecular qubits for quantum information technologies.^19^ Very large HFCs may be obtained if the PSO interaction is small and the FC term is large.^20,21^ This can lead to so-called “clock transitions” and an increase in phase memory time,^21^ as such SMMs are less sensitive toward quantum decoherence.^22,23^

To support the interpretation of the EPR spectra or the in silico design of magnetic materials such as SMMs, quantum-chemical methods are of great importance. However, the accurate description of the electronic structure of open-shell systems is a complicated task. Various methods from complete or restricted active space self-consistent field (CASSCF/RASSCF) approaches to coupled-cluster methods and density functional theory (DFT) are routinely applied to EPR properties, see, e.g., refs (24–29). For large systems, the latter is the method of choice in terms of feasibility. Unfortunately, the choice of the density functional approximation (DFA) is a nontrivial task, as the performance of a given DFA may be highly dependent on the given molecule or nucleus.^9,30−36^

Over the past two decades, the adiabatic connection random phase approximation^37−39^ (RPA) has emerged as a useful tool in quantum chemistry and materials science.^40−76^ As shown in previous works and exploited here, a practically useful expression for the correlation energy is derived by combining the adiabatic connection and the fluctuation dissipation theorem.^38,39,77^ Using the adiabatic connection, a noninteracting system can be related to the physical many-particle system using a coupling strength parameter. The latter varies from 0 to 1, i.e. from the noninteracting to the fully interacting system. The correlation energy can subsequently be formulated as a coupling strength integral, with the integration boundaries accordingly ranging from 0 to 1. In its simplest but most successful form, the integrand is related to pure Coulomb interactions, i.e. neglecting exchange and higher-order correlation terms, leading to the so-called direct random phase approximation (dRPA). For a more detailed outline of the theory, we refer to the noted references. From a physical point of view, the RPA correlation energy can be interpreted as a zero-point vibrational energy difference of harmonic oscillators. Here, the oscillators correspond to electronic excitations,^41,44^ which relates modern RPA to the original formulation of plasma oscillations in a high-density electron gas.^37^ Alternatively, it has been shown that the RPA can also be derived from the ring coupled-cluster doubles approximation.^45^

Today, RPA methods can be employed in a post-Kohn–Sham framework based on converged Kohn–Sham orbitals^40−61^ or in a self-consistent fashion to account for the density-driven error.^69−75^ Analytical derivatives for chemical properties were formulated for both frameworks.^78−82^ Alternatively, the RPA correlation energy can be used as a functional ingredient in the context of double hybrid functionals^83^ or σ-functionals.^84−91^ Especially the latter class of functionals has received recent interest.

Already the simplest approximation, i.e. dRPA, shows many attractive features.^60,61^ Although dRPA correlation energies show a dependence on the underlying KS reference, they are otherwise free from empiricism. When using nonempirical KS functionals such as the generalized gradient approximation (GGA) PBE^92^ or the meta-generalized gradient approximations (*meta*-GGAs) TPSS^93^ and r^2^SCAN^94,95^ an accurate first-principles DFT method is obtained. Second, it achieves similar accuracy as hybrid functionals without the need to compute Hartree–Fock (HF) exchange in each self-consistent field (SCF) iteration. When RPA is applied in a post-Kohn–Sham framework, the SCF procedure is solved with a semilocal or hybrid DFA, and the RPA correlation energy and HF exchange energy are computed only once. Third, RPA is applicable to small-gap systems which is a distinctive feature over other post-KS or post-HF approaches.

So far, the performance of RPA was mainly assessed for energies and geometry properties.^61^ Magnetic properties are comparably unexplored. Notable studies in this regard are the application of dRPA to nuclear magnetic resonance (NMR) shieldings and shifts,^82,88^ as well as its application to finite magnetic fields.^57,96^ Given the success of RPA in quantum chemistry, further studies on the performance of RPA for magnetic properties such as EPR parameters are clearly desirable.

In this work, we assess the accuracy of the dRPA as a post-Kohn–Sham method for the FC and the SD HFC terms. This is done in a nonrelativistic and in the scalar-relativistic exact two-component^97−99^ (X2C) framework. The results are intended to serve as a first test across the periodic table of elements and help to guide future research directions for accurate predictions of EPR properties.

## Computational Methods

### Theory

The FC and SD terms can be evaluated as expectation values, i.e. the matrix representation of the operators is contracted with the density matrix in the atomic orbital (AO) basis. In a nonrelativistic framework, these HFC contributions in atomic units read^10,13^
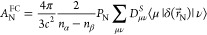
1
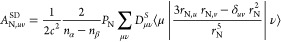
2where  denotes the Dirac delta distribution, δ_*uv*_ the Kronecker delta, and *D*_μν_^*S*^ the AO spin excess density matrix element of the basis functions μ, ν. *c* is the speed of light and *u*, *v* are the Cartesian directions. *n*_α_ and *n*_β_ is the number of α and β electrons, respectively.  denotes the electron–nucleus position vector and *r*_N_ refers to the norm. *P*_N_ = β_e_*g*_e_β_N_*g*_N_ collects the electron and nuclear *g*-factors *g*_e_ and *g*_N_, as well as Bohr’s magneton β_e_, and the nuclear magneton β_N_. With standard Kohn–Sham methods, the one-particle density matrix is available from the eigenvectors
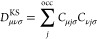
3where σ denotes the spin, μ, ν the AO basis functions, and *j* the Kohn–Sham eigenstates. Here, the coefficients *C*_μj_ of the ground-state calculation are real, and only occupied (occ) orbitals are included in the summation. For post-Kohn–Sham methods, the KS density matrix **D**^KS^ is changed due to electron correlation and orbital relaxation contributions. Thus, the RPA density matrix reads
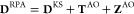
4

Here, **T**^AO^ is the unrelaxed one-particle AO density matrix due to RPA correlation and **Z**^AO^ is the relaxation-only one-particle AO density matrix.^79^ This relaxed density matrix is also needed for RPA geometry gradients^78−80^ and we briefly review its calculation with the resolution of the identity (RI) approximation as previously^79^ implemented in the TURBOMOLE program suite.^100−104^

The RI-RPA correlation energy is defined with an imaginary frequency integration according to^42^
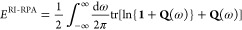
5where all quantities are calculated in the RI auxiliary space. The matrix **Q** corresponds to a single ring diagram in the coupled-cluster framework^42,45^ and is defined as
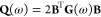
6with the three-index matrix
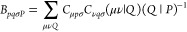
7

Here, *p*, *q* denote general KS molecular orbitals (MOs) and *P*, *Q* the RI auxiliary basis functions. We use Mulliken notation for the electron repulsion integrals. **B** is also known from other methods such as second-order Møller–Plesset perturbation theory^105^ (MP2) or CC2.^106,107^ The remaining matrix **G** in the MO space is given as
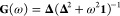
8with the energy-dependent matrix
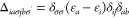
9*i*, *j* refer to occupied KS orbitals, whereas *a*, *b* refer to virtual KS orbitals. ε are the KS energy eigenvalues.

The density correction **T** is evaluated in the MO space as
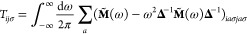
10
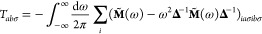
11**M̃** is a symmetric supermatrix defined as
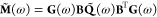
12
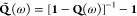
13

Note that the occupied-virtual and virtual-occupied block is zero due to missing relaxation terms. The calculation of this unrelaxed density correction is the most time-consuming step of an RI-RPA gradient and HFC calculation, as it asymptotically scales with . *N* measures the size of the system. It is also the most demanding part in terms of memory and disk storage.^79^ For the RPA density matrix, **T** is transformed to the AO space.

Orbital relaxation effects are included by solving the coupled-perturbed Kohn–Sham (CPKS) equation
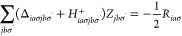
14

Therefore, the relaxed density matrix **Z** only contributes to the occupied-virtual and virtual-occupied MO tensor space. The required matrix **H**^+^ generally reads
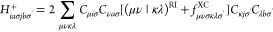
15

**H**^+^ includes the two-electron Coulomb integral in the RI approximation^108−110^ (RI-J) and the exchange–correlation (XC) kernel *f*^XC^ of the underlying KS reference in the adiabatic approximation. For hybrid functionals, the XC kernel includes a fraction of exact exchange. The CPKS right-hand side **R** is given as
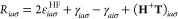
16where **ε**^HF^ is obtained by calculating the Fock matrix at the converged KS orbitals. Here, the occupied-virtual block of the HF matrix in the MO space is nonzero, as the KS orbitals are not generally eigenfunctions of the Fock operator. Finally, the matrix γ is defined as
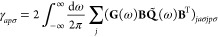
17
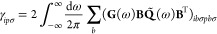
18and the right-hand side can be accumulated. Then, only **Z** is left to be determined iteratively. Here, the single CPKS equation in eq 14 is solved similarly as for excited-state properties.^111^ The solution vector **Z** is subsequently transformed to the AO space to construct the RPA density matrix. Compared to the preceding calculation of the unrelaxed density correction **T**, this step is computationally inexpensive. Overall, the calculation of the HFC and other properties with the RI-RPA method^79^ is computationally less demanding than RI-MP2^105^ and RI-CC2 calculations,^106,107^ which scale as .

For an existing RPA gradient implementation, only the HFC matrix needs to be interfaced into the RPA module. This holds for both the nonrelativistic and the scalar-relativistic framework, as the latter only affects the HFC matrix.

### Computational Settings

First, we consider the test sets 1 (small main-group compounds) and 2 (large main-group compounds) of the Bartlett group described in ref (30). Structures are taken from the literature and the same basis sets as in the original benchmark study are applied to allow for a consistent comparison to the coupled-cluster theory with singles, doubles, and perturbative triples CCSD(T). We omitted the Be compounds of test set 1 and Zn-porphycene of test set 2 in the present work, as this simplifies the basis set and auxiliary basis set settings. The aug-cc-pVTZ-J basis set,^112−114^ taken from the Basis Set Exchange (BSE) library,^115−118^ is employed for all elements. To cover the most important rungs of Jacob’s ladder in DFT,^119,120^ we apply the pure functionals BP86,^121,122^ BLYP,^121,123^ PBE,^92^ TPSS,^93^ and r^2^SCAN^94,95^ as well as the global hybrids PBE0,^124^ TPSSh,^125^ and r^2^SCANh.^126^ For PBE, the range-separated hybrid LC-ωPBE^127^ and the local hybrid LH14t-calPBE^128^ are further employed. The latter class is additionally represented by LH20t^129^ and TMHF.^33^ Libxc is applied for r^2^SCAN, r^2^SCANh and LC-ωPBE.^130−132^ All local hybrid functionals make use of a seminumerical integration scheme.^133,134^ Note that BLYP, PBE, TPSS, PBE0, and TPSSh were already included in the original study of ref (30). We consider the semilocal functionals BP86,^121,122^ BLYP,^121,123^ PBE,^92^ TPSS,^93^ and r^2^SCAN^94,95^ as KS reference for the RPA calculations. Additionally, RPA calculations are performed with a PBE0 and an HF reference solution, as especially the latter option performed very well for NMR shieldings and shifts.^88^ Large integration grids are used (grid size 5a without pruning).^135−137^ For comparison HF, MP2, and CC2 calculations are carried out. For MP2 and CC2, the scaled same-spin and scaled opposite-spin (SOS) variants are also applied with the standard factors.^138,139^ RPA,^42^ MP2,^105^ and CC2^106^ make use of the RI approximation with the aug-cc-pV6Z-RIFIT auxiliary basis sets^104,140^ taken from the BSE library.^115−118^ Additionally, the RPA calculations make use of the RI-J approximation for the calculation of the HF energy with the universal auxiliary basis sets.^141^ The RI approximation is not applied for the SCF procedure, which is converged with tight thresholds of 10^–8^ Hartree for the energies and 10^–7^ for the root-mean-square of the density matrix change. For MP2 and CC2, the threshold for the norm of the residual vector in the solution of the Z vector equations is set to 10^–6^. The imaginary frequency integration for RPA is carried out with the Gauss–Legendre method and 120 integration points. We note in passing that 80 points are already sufficient for converged HFC constants. All HF, MP2, CC2, semilocal and hybrid DFT, as well as RPA calculations herein are performed with TURBOMOLE^100−104^ for maximum consistency. Further, calculations with the DSD-PBEP86 double hybrid functional^142,143^ are performed with ORCA Version 5.0.4.^144,145^ Here, the RI-J approximation, with the universal auxiliary basis sets^141^ (called def2/J within ORCA) for the DFT part and the aug-cc-pV6Z-RIFIT basis set taken from the BSE library for the MP2 part, and the chain of spheres (COSX) approximation are applied.^146,147^ An SCF threshold of 10^–8^ Hartree is chosen and the integration grids are chosen in accordance with ref (30) (IntAcc = 6, AngularGrid = 7). The frozen core approximation is not employed throughout this work. For the evaluation of diethylaminyl, we chose to evaluate H6, H7, H10, H11 as one data point due to the symmetry of the molecule instead of splitting them up into two as done in ref (30). Throughout this work, we list the results in MHz.

Second, we apply the RPA approach to a subset (ScO, TiF_3_, MnF, MnO_3_, Mn(CO)_5_, Fe(CO)_5_^+^) of the transition metal complexes described in ref (36). The structures of these complexes are optimized with the def2-TZVP basis set^148^ and the TPSS functional^93^ using a large grid (grid size 5 with pruning).^135,136^ Derivatives of quadrature weights are included in the calculations. The D4 dispersion correction^149^ is applied for the calculations, as well as the RI-J approximation with the def2-TZVP auxiliary basis set.^141^ Tight convergence thresholds of 10^–8^ Hartree are chosen for the energies. For the structure optimization, the default convergence criteria of 10^–6^ Hartree and 10^–3^ Hartree/bohr are chosen. The optimizations are done within the following point group symmetries. TiF_3_ is optimized within D_3h_ symmetry, MnO_3_ within C_3v_ symmetry, and Mn(CO)_5_ as well as Fe(CO)_5_^+^ within C_4v_ symmetry. ScO and MnF were optimized without symmetry constraints. We note in passing that the experimental reference postulates D_3h_ symmetry for MnO_3_.^150^ However, this symmetry is not obtained with the TPSS functional, but with the PBE0 functional. The wave function with PBE0 depicts strong spin-contamination with ⟨*S*^2^⟩ = 0.97, which is why we chose to use the TPSS result. The calculations of the HFC constants are done with the same parameters and the same methods, excluding the DSD-PBEP86 functional, as for the test sets 1 and 2 of the Bartlett group. Only the auxiliary basis for RPA, MP2, and CC2 is changed. The aug-cc-pV6Z-RIFIT auxiliary basis sets are only used for the light atoms and the aug-cc-pV5Z-RIFIT auxiliary basis sets^151^ taken from the BSE library^115−118^ are used for the metal atoms because there is no aug-cc-pV6Z-RIFIT basis set available for these elements. The calculation of the HF energy for RPA is still done with the RI-J approximation and the universal auxiliary basis.^141^ The symmetries of the molecules are not exploited for the HFC calculations.

Third, we apply the RPA approach to lanthanide SMMs with large HFC constants, namely [La(OAr*)_3_]^−^, [Lu(NR_2_)_3_]^−^, and [Lu(OAr*)_3_]^−^ with OAr* = 2,6-Ad_2_-4-*t*-Bu-C_6_H_2_O, Ad = adamantyl, *t*-Bu = *tert*-butyl, R = SiMe_3_ with Me = methyl.^21^ To account for relativistic effects, we use the scalar X2C Hamiltonian in the diagonal local approximation to the unitary decoupling transformation (DLU),^152−154^ as spin–orbit effects were shown to be small for these compounds.^18,32,155,156^ A finite nucleus model with a Gaussian charge distribution^157^ is applied for the scalar potential and the vector potential.^158,159^ The x2c-TZVPall-2c basis set is applied for the lanthanide atoms, while the x2c-SVPall-2c basis set is used otherwise.^160^ We use large grids (grid size 4a without pruning) for the numerical integration.^135−137^ The PBE,^92^ TPSS,^93^ r^2^SCAN^94,95^ PBE0,^124^ TPSSh,^125^ r^2^SCANh,^126^ LC-ωPBE,^127^ and LH14t-calPBE^128^ functionals are considered. Based on the previous results, HF and RPA@HF are not applied for the lanthanide systems. The SCF procedure is converged with tight thresholds of 10^–8^ Hartree for the energies and 10^–7^ for the root-mean-square of the density matrix change. The conductor-like screening model^161,162^ (COSMO) is applied with the parameters for tetrahydrofuran (permittivity of 7.52) and the default radii are applied (La = 2.2230, Lu = 2.2230, C = 2.0000, O = 1.7200, H = 1.3000, Si = 2.2000, N = 1.8300; all radii in Ångström). The RI-J approximation is only used for the RPA calculations with a tailored fitting basis set (see Supporting Information). The calculation of the HF energy for RPA is done with the RI-J approximation and the x2c-universal auxiliary basis.^160,163^ Principal components of the HFC tensor are obtained from the symmetric form of the tensor. Computationally optimized structures are taken from the literature.^21^ Currently, the RPA relaxed density is computed without COSMO.^79^ We estimate the impact of this error by performing semilocal DFT and RPA calculations without COSMO throughout, see Supporting Information. Further estimates are based on HF and RI-MP2 calculations, i.e. HF(no-COSMO)/MP2(no-COSMO), HF(COSMO)/MP2(no-COSMO), and HF(COSMO)/MP2(COSMO) calculations are carried out for [Lu(NR_2_)_3_]^−^, as this compound shows the largest effects of COSMO at the DFT level.

## Results and Discussion

### Small Main-Group Systems

**Figure 1:**
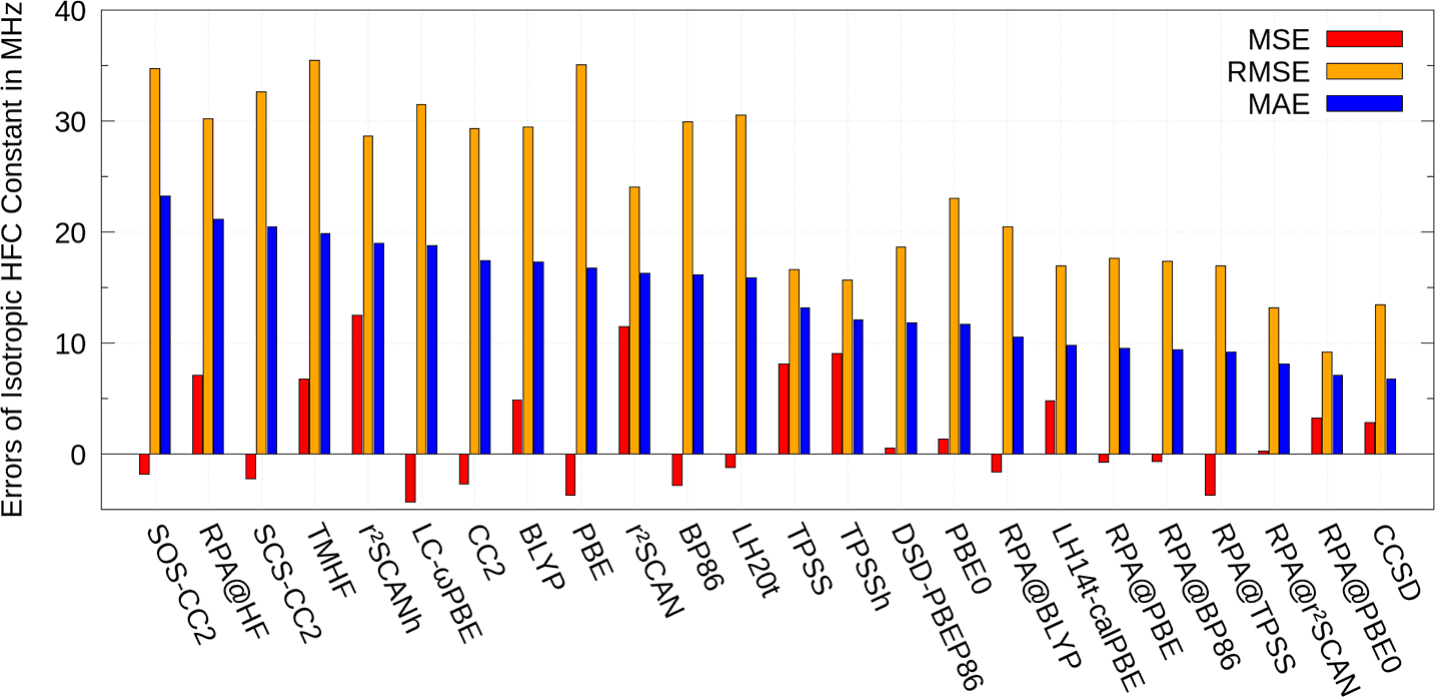
Assessment of accuracy and statistical evaluation of various DFT and post-HF methods for the isotropic hyperfine coupling constants of the test set 1 of ref (30) consisting of 23 small main-group radials. The MSE, RMSE, and MAE are shown. Deviations are measured with respect to CCSD(T) results in MHz. RPA@BLYP denotes that the RPA correlation energy and density are computed at the BLYP Kohn–Sham solution, the same holds for other functionals. Results with HF and MP2 are omitted in this figure, as large errors are observed with these methods. CCSD results are taken from ref (30). Individual results and spin expectation values are listed in the Supporting Information. The set includes 22 ^1^H, 2 ^11^B, 17 ^13^C, 4 ^14^N, 8 ^17^O, 1 ^19^F, 1 ^31^P, 2 ^33^S, and 1 ^35^Cl chemically inequivalent nuclei.

A set of small main-group systems is considered first, as high-level CCSD(T) results are available. It was shown that CCSD(T) performs excellently for the HFC of the given test set.^30^ When neglecting spin–orbit effects and the PSO term, the isotropic HFC constant consists of the FC term, as the SD term only affects the anisotropy and the principal components. Thus, the comparison to CCSD(T) results essentially assesses the accuracy of the spin excess density at the respective nuclei. The mean signed errors (MSE), mean absolute errors (MAE), and root-mean-square errors (RMSE) for the test set composed out of the small main-group systems with respect to the CCSD(T) results for the HFC constants in ref (30) are shown in Figure 1. RPA can be easily included in the accuracy ordering of ref (30) right behind the coupled-cluster approaches. That is, the quality of the HFC constants for small main-group radicals follow the ordering CCSD > RPA@DFA > Hybrid DFAs > Semilocal DFAs > CC2 > MP2 > HF. In contrast, a detailed ordering of the hybrid DFAs with global, range-separated, and local hybrids is difficult, as already observed in ref (30) for GGA-based global hybrids, *meta*-GGA-based global hybrids, and range-separated hybrids. Removing nuclei with an HFC of more than 1000 MHz from the test set leads to essentially the same ordering, see Supporting Information.

Turning toward the DFT treatment in detail, the MAEs are clearly reduced and especially RPA@DFA performs excellently. Five of the six employed RPA@DFA approaches produce the five lowest MAEs. Only the local hybrid LH14t-calPBE leads to a lower MAE of 9.7 MHz in comparison to the “worst” RPA@DFA approach, namely RPA@BLYP. Additionally, the MAEs of RPA@DFA are remarkably close together and span a range of 7.1 MHz (RPA@PBE0) to 10.5 MHz (RPA@BLYP). In comparison, the MAEs for the corresponding pure functionals already span a range from 13.1 MHz (TPSS) to 17.3 MHz (BLYP) and the MAE of the hybrid functional PBE0 amounts to 11.7 MHz. However, this does not necessarily hold for all individual data points. Here, the GGA-based RPA results tend to be rather close to each other, while the *meta*-GGA-based results might deviate more. For instance, the HFC constants of CH are described very differently with RPA@TPSS compared to the other RPA@DFA approaches. The global hybrids span a range of 11.7 MHz (PBE0) to 18.9 MHz (r^2^SCANh), whereas the PBE-based range-separated hybrid LC-ωPBE leads to an MAE of 18.8 MHz. The three local hybrids are very far apart with an MAE of 9.7 MHz for LH14t-calPBE, 15.9 MHz for LH20t and 19.9 MHz for TMHF. Thus, RPA leads to the most notable improvement upon PBE and the admixture of exact exchange with global hybrids, range-separation, as well as a fully local admixture is inferior in this regard.

With respect to the RMSE, the RPA@DFA results are a somewhat more spread out and span a range of 9.2 MHz (RPA@PBE0) to 20.5 MHz (RPA@BLYP). This makes RPA@PBE0 the best DFT method in comparison to CCSD(T) for the small test set, as it produces both the lowest MAE and the lowest RMSE. RPA@r^2^SCAN ranks second. Functionals without exact exchange produce RMSEs from 16.6 MHz (TPSS) to 35.0 MHz (PBE), global hybrids from 15.7 MHz (TPSSh) to 28.7 MHz (r^2^SCANh), and local hybrids from 17.0 MHz (LH14t-calPBE) to 35.5 MHz (TMHF).

The excellent performance of RPA@DFA is even more remarkable when comparing it to MP2 and CC2 which come with increased computational demands. HF and the MP2 methods lead to large MAE (ranging from 38.5 MHz for MP2 to 80.2 MHz for HF) and RMSE values (ranging from 57.9 MHz for MP2 to 111.3 MHz for HF). The different CC2 approaches lead to MAEs from 17.4 MHz (CC2) to 23.2 MHz (SOS-CC2) and to RMSEs from 29.3 MHz (CC2) to 34.7 MHz (SOS-CC2). Therefore, reliable post-HF results already require a very expensive treatment of electron correlation with at least CCSD. Notably, the MP2-based double hybrid DSD-PBEP86 functional performs much better than MP2 and CC2 and very similar to PBE0, but it is still outperformed by the RPA@DFA approaches.

We note that the performance of r^2^SCAN observed in the present work is in striking contrast to the behavior found for its parent SCAN^164^ in ref (30). This can be rationalized by the pronounced grid sensitivity of SCAN,^94,165,166^ which is especially detrimental for properties depending on the density in the vicinity of the nuclei. In line with our previous work on magnetic properties,^156,167−170^ r^2^SCAN is a rather stable and robust functional. Therefore, we recommend to only use r^2^SCAN and not SCAN for EPR and RPA calculations.

Overall, the RPA@DFA approaches produce good results with respect to both the MAEs and the RMSEs. Especially RPA@r^2^SCAN and RPA@PBE0 perform almost as good as CCSD. In comparison to established hybrid functionals, the results are of similar quality or even better. Additionally, the median RPA results are not notably reliant on the chosen DFA as starting point. All semilocal DFA starting points lead to very similar results, especially compared to the rather broad span of the results with semilocal DFT. Also, the deviations between RPA@PBE and RPA@PBE0 are smaller than that of PBE and PBE0. Thus, RPA alleviates the difficult choice of finding the “right” DFA.

### Large Main-Group Systems

**Figure 2:**
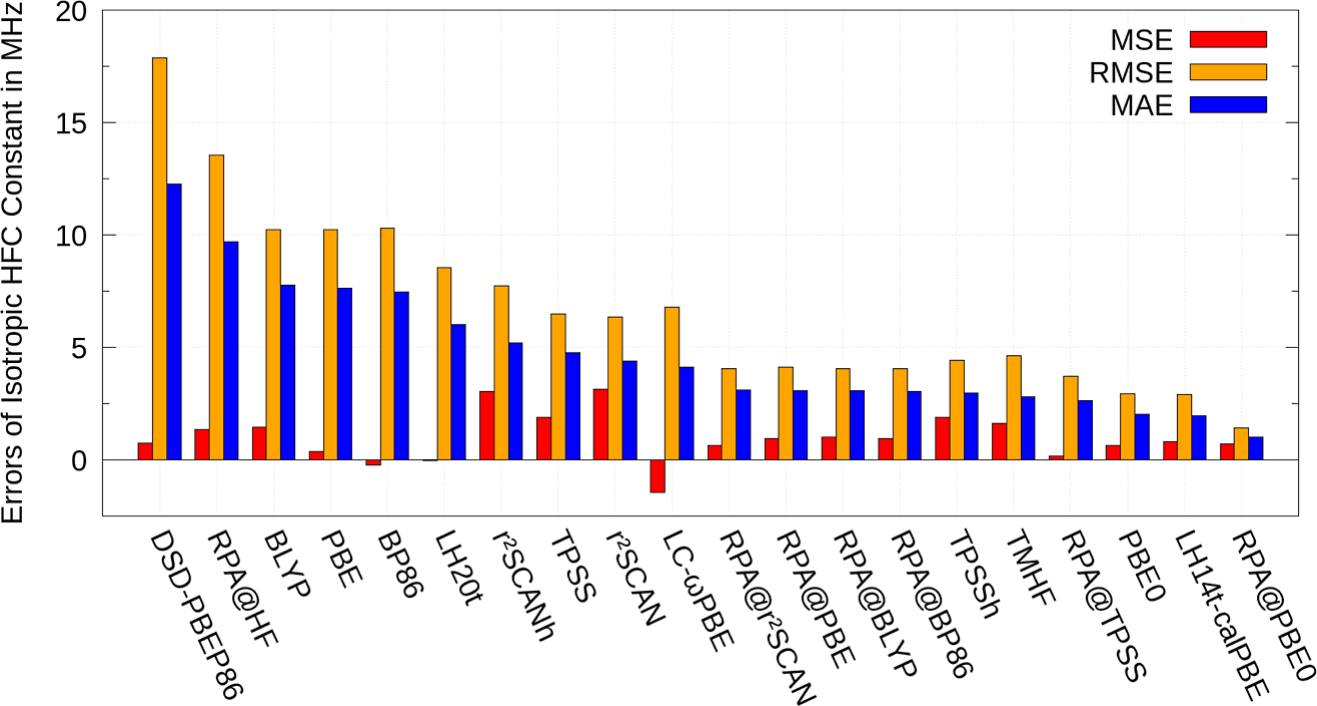
Assessment of accuracy and statistical evaluation of various DFT methods for the isotropic hyperfine coupling constants of test set 2 of ref (30) consisting of 8 large main-group systems. The MSE, RMSE, and MAE are shown. Deviations are measured with respect to CCSD results in MHz. RPA@BLYP denotes that the RPA correlation energy and density are computed at the BLYP Kohn–Sham solution, the same holds for other functionals. Results with HF, MP2, and CC2 are omitted in this figure, as large errors are observed with these methods. Individual results and spin expectation values are listed in the Supporting Information. The test set includes 33 ^1^H, 32 ^13^C, 6 ^14^N, 1 ^17^O, and 1 ^33^S chemically inequivalent nuclei.

The MSEs, MAEs, and RMSEs for the test set composed out of large organic systems with respect to the CCSD results for the HFC constants in ref (30) are shown in Figure 2. Results for all employed methods are depicted, except for HF, MP2 and CC2 methods because of large MAEs, ranging from 17.6 MHz for CC2 to 44.1 MHz for HF. The same holds for the RMSE values, ranging from 24.8 MHz for CC2 to 58.2 MHz for HF. Again, the DSD-PBEP86 double hybrid performs much better than MP2 and CC2, as it results in an MAE and RMSE of 12.3 and 17.9 MHz, respectively. Note that the MAEs and RMSEs for this test set are generally smaller, which is at least partly caused by the overall smaller values of the calculated HFC constants.

RPA@PBE0 performs best with an MAE of less than 1 MHz. The PBE based local hybrid LH14t-calPBE features the second lowest MAE with a value of 2.0 MHz, while LH20t gives the largest MAE of 6.0 MHz among the considered local hybrids. For both test sets, LH20t and TMHF are less robust than LH14t-calPBE. Global hybrids span a range from 2.0 MHz (PBE0) to 5.2 MHz (r^2^SCANh) and the pure functionals result in MAEs from 4.4 MHz (r^2^SCAN) to 7.8 MHz (BLYP). The range-separated LC-ωPBE leads to a mean absolute error of 4.1 MHz. Just like for the first test set, the admixture of exact exchange worsens the performance of r^2^SCAN, while application of the RPA upon the semilocal DFA leads to an improvement.

The RMSE results are similar to the MAE results. For the RPA approaches with semilocal DFAs, the RMSEs appear in a very close range from 3.7 MHz (RPA@TPSS) to 4.1 MHz (RPA@PBE), while the RMSE of RPA@PBE0 amounts to 1.4 MHz. Results for the local hybrids range from 2.9 MHz (LH14t-calPBE) to 8.5 MHz (LH20t). For the global hybrids they range from 2.9 MHz (PBE0) to 7.7 MHz (r^2^SCANh) and for the pure functionals they are in the region of 6.4 MHz (r^2^SCAN) to 10.3 MHz (BP86). LC-ωPBE leads to an RMSE of 6.8 MHz. Therefore, the order of accuracy according to RPA > Hybrid DFAs > Semilocal DFAs > CC2 > MP2 > HF is also valid for the test set with larger molecular systems.

To sum up the results of test sets 1 and 2, the RPA@DFA approaches produce very good MAE and RMSE values in comparison to the other considered methods. This holds for all tested KS starting points. Notably, also LH14t-calPBE, PBE0, and TPSSh produce very good results for both test sets. The robust performance of PBE0 and TPSSh was already observed in the original study of ref (30). Concerning the range of the results for each rung of Jacob’s ladder, RPA outperforms the semilocal and hybrid functionals. Additionally, RPA also clearly outperforms MP2 and CC2 representing post-HF methods—although these come with increased computational costs compared to RPA.

### Transition-Metal Systems

In order to test whether the RPA approach can lead to good results for transition-metal systems, which are often studied with EPR experiments, a subset of the compounds investigated in ref (36) is considered. That is, small molecules with a known HFC constant are studied. The electronic structure of ScO, TiF_3_, MnO_3_, Mn(CO)_5_, and Fe(CO)_5_^+^ is made up of one unpaired electron, whereas that of MnF includes six unpaired electrons.

In Table 1, the calculated HFC constants of the 3d transition metals within those compounds are compared to experimental values,^150,171−175^ which were collected in ref (36). Only the results for the DFT based approaches are shown. MP2 and CC2 results are listed in the Supporting Information as these methods perform poorly. As expected, especially MP2 leads to very large errors.

**Table 1:**
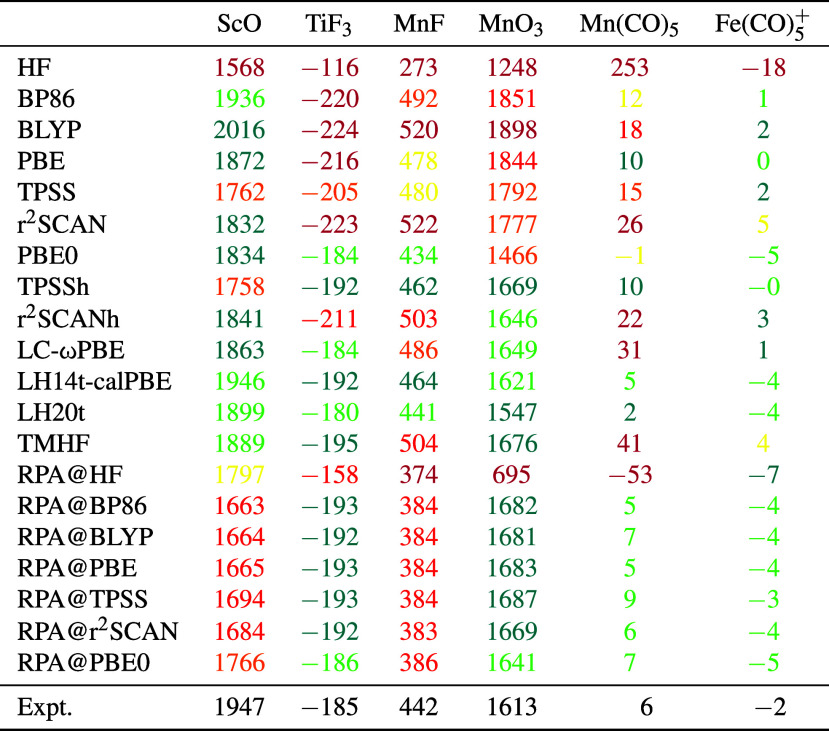
Isotropic Hyperfine Coupling Constants (in MHz) of 3d Transition Metals within Small Compounds Using Various DFT Methods and Comparison to the Experimental Findings (Expt.) as Collected in ref (36)^a^

a MP2 and CC2 results are only given in the Supporting Information. For ScO, TiF_3_, MnF, and MnO_3_, a color code is used based on the percent-wise deviation of the calculated HFC constant towards the experimental findings: green (less than 3%), teal (less than 6%), yellow (less than 9%), orange (less than 12%), red (less than 15%), purple (more than 15%). For Mn(CO)_5_ and Fe(CO)_5_^+^, the colors are assigned for absolute values in MHz: green (less than 3 MHz), teal (less than 6 MHz), yellow (less than 9 MHz), orange (less than 12 MHz), red (less than 15 MHz), purple (more than 15 MHz).

As observed for the first two test sets, the different RPA results based on semilocal DFAs are quite similar to each other and relatively independent of the chosen DFA. Results with RPA@PBE0 again deviate somewhat more from the other RPA approaches. The agreement with experiment is generally very good, with the exception of ScO and MnF. For ScO, the absolute deviations for the RPA@DFA approaches range from 181 MHz (RPA@PBE0) to 284 MHz (RPA@BP86) or from around 9 to 15%. This is generally larger than for the other methods considered herein. The deviations of around 13% from the experimental value for MnF are also among the larger observed deviations for the considered DFT approaches. However, the RPA@DFA approaches work particularly well for the small HFC constants of Mn(CO)_5_ and Fe(CO)_5_^+^. Here, the correct order of magnitude and the sign of the experiment is reproduced.

Additionally, the overall best results are obtained by LH14t-calPBE, which is in very good agreement for all of the considered experimental values. LH20t is also in very good agreement with experiment. Both give a correct description of the two small constants on Mn(CO)_5_ and Fe(CO)_5_^+^. The other considered functionals give generally reasonable results with varying degrees of accuracy in terms of absolute values. A weak point is a good description of the small constants on Mn(CO)_5_ and Fe(CO)_5_^+^. Often, one of the signs is wrong or the HFC constant on Mn(CO)_5_ is too large in relative terms. Except for the RPA@DFA approaches, LH14t-calPBE, and LH20t, only TPSSh leads to good results for both small HFC constants.

Overall, RPA@DFA performs well for central atom’s HFC of the considered transition-metal compounds. The results only clearly fall behind the very good agreement with experiment for LH14t-calPBE and LH20t. This is mainly due to larger deviations for ScO and MnF. The decisive point of the RPA approaches is again the relative independence of the results on the chosen DFA starting point. Additionally, the RPA approaches allow for a good description of the two small HFC constants. However, the set of considered molecules is relatively small and the PSO term needs to be generalized to RPA for broad applicability among transition-metal systems. Currently, the PSO term would have to be approximated with the KS reference.

### Lanthanide SMMs

In Table 2, calculated values for the principal components and the isotropic HFC constants of the three lanthanide SMMs [La(OAr*)_3_]^−^, [Lu(NR_2_)_3_]^−^, and [Lu(OAr*)_3_]^−^ are compared to the experimental findings of ref (21). These molecules show very large HFC constants and [La(OAr*)_3_]^−^ and [Lu(OAr*)_3_]^−^ consist of more than 200 atoms. Therefore, these complexes serve as an example for extended systems with a pronounced spin excess density at a heavy nucleus.

**Table 2:**
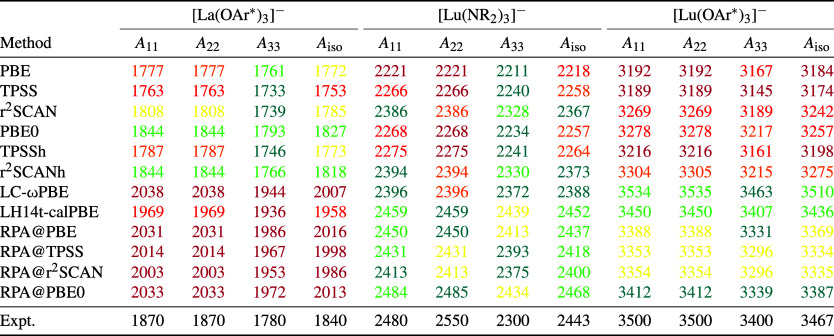
Principal Components of the HFC-Tensor and the Isotropic Constant *A*_iso_ (in MHz) for the Three Spin-1/2 La(II) and Lu(II) Molecules [La(OAr*)_3_]^−^, [Lu(NR_2_)_3_]^−^, and [Lu(OAr*)_3_]^−^ with the Scalar-relativistic DLU-X2C Hamiltonian and the x2c-TZVPall-2c/x2c-SVPall-2c Basis Set^a^

a Experimental results (Expt.) are taken from ref (21). Spin expectation values are listed in the Supporting Information. The experimental uncertainties for the principal components and the isotropic constants are ±25 MHz for [La(OAr*)_3_]^−^ and ±50 for [Lu(NR_2_)_3_]^−^ as well as [Lu(OAr*)_3_]^−^. We use the following color code to illustrate the accuracy of the computed results compared to the experimental findings within the range of the experimental uncertainties (25 or 50 MHz). Green (within the experimental range), teal (deviation to the experiment is within two times the experimental uncertainty), yellow (with three times the experimental uncertainty), orange (four times), red (five times), purple (six times and more).

For the lanthanide systems, we broaden our view from the HFC constants to the principal components of the tensors, as these demonstrate the axial symmetry of the large molecules. Note that we only show scalar-relativistic results here. Due to the large s character of the unpaired electron,^21^ the FC term dominates the HFC constant.^32^ Therefore, a scalar-relativistic treatment is sufficient for the HFC constant, as shown in refs (18) and (32). Matters are different for lanthanide systems with open f shells or more unpaired electrons.^16,25,32,156^ Then, inclusion of spin–orbit coupling is key to accurate results.

Looking first at the principal components, the axial symmetry of the principal components is obtained with all methods. PBE severely underestimates the difference of *A*_11_ and *A*_22_ to *A*_33_, whereas RPA@PBE and the hybrids alleviate this situation. As observed before, the RPA results span a smaller range than the pure DFA results they are based on. Especially, r^2^SCAN deviates more notably from PBE and TPSS. Both RPA and the admixture of exact exchange leads to a very consistent increase of all principal components and consequently the isotropic HFC constants. Therefore, RPA and hybrids clearly lead to an improvement for the Lu compounds, as semilocal DFAs such as PBE and TPSS substantially underestimate the HFC. Here, RPA@PBE0 performs best among the RPA methods. For the La complex, semilocal DFAs already lead to rather good agreement with the experiment. Hybrid functionals such as PBE0 and r^2^SCANh then perform very well. Here, RPA or range-separated and local hybrids result in too large HFCs.

The overall best results for the isotropic HFC constants of all three molecules are obtained for r^2^SCANh and LH14t-calPBE. For the Lu complexes, the results of LH14t-calPBE are within the experimental uncertainties. The PBE-based functionals show that the HFC is very sensitive toward the detailed admixture of exact exchange. RPA@PBE leads to results roughly in the range of the three hybrids PBE0, LC-ωPBE, and LH14t-calPBE. Additionally, the RPA results of the different KS starting points are again very close together. The deviations are overall in the same region as for the other DFT approaches and the agreement with experiment is reasonable.

As noted in the computational settings, COSMO is not yet supported for the calculation of the relaxed RPA density matrix. Thus, it is only used for the KS reference calculation. Completely neglecting COSMO throughout changes the isotropic HFC constant of the three SMMs by 5, 3, and 5 MHz at the RPA@PBE level. The results with PBE are altered by −4, 60, and −19 MHz, respectively. As a further estimate of the error by neglecting COSMO for the RPA part, we consider results from MP2 and [Lu(NR_2_)_3_]^−^. Here, a full treatment of COSMO leads to an isotropic HFC constant of 2474 MHz. Using COSMO for the HF part only yields 2542 MHz and fully neglecting COSMO results in 2589 MHz. For comparison, the difference in the results for the scalar-relativistic treatment and the full two-component spin–orbit approach amounts to −16, –31, and −14 MHz for the three compounds at the PBE0/x2c-QZVPall-2c level, which is smaller than the experimental uncertainties.^32^ Therefore, we do not expect major changes by a full inclusion of COSMO for the relaxed RPA density matrix of the three SMMs. Matters may be different for highly charged systems.

In terms of computational costs, the RPA calculations of the larger complexes [La(OAr*)_3_]^−^ and [Lu(OAr*)_3_]^−^ take roughly 1 day on a central processing unit of type Intel Xeon Gold 6212U at 2.40 GHz with 24 threads or about 2 days with 12 threads (shared memory parallelization with Open Multi-Processing). Overall, RPA in its post-KS fashion is easily applicable to extended systems when using the resolution of the identity approximation.^42,79^

To sum up, it is demonstrated that the RPA approach also works on large lanthanide systems with a spin excess density with pronounced s character. This was demonstrated both for the principal components of the HFC tensor and the isotropic HFC constant. In comparison to the other considered DFT methods, the RPA calculations do not result in the best agreement with experiment overall, but are generally on the same level as hybrid functionals. In a direct comparison between the RPA methods and the corresponding pure functionals for the HFC constants, the deviations for [La(OAr*)_3_]^−^ were smaller with the respective KS reference, while the RPA results lead to a smaller deviation from experiment for [Lu(NR_2_)_3_]^−^ and [Lu(OAr*)_3_]^−^.

## Conclusion

Our results show that RPA performs remarkably well for HFC constants and tends to substantially improve upon its KS reference. It clearly outperforms post-HF methods such as MP2 and CC2, while coming with reduced computational demands. Notably, the RPA results only show a minor dependence on the KS reference. Moreover, RI-RPA is applicable to large molecules as shown for the lanthanide SMMs with more than 200 atoms. This means that RPA is expected to become a useful tool for the study of EPR HFC constants.

Extensions of the described framework are possible in multiple directions, namely the inclusion of the PSO term, extension to the class of σ-functionals, or the generalization to self-consistent RPA methods.

## Data Availability

The data that support the findings of this study are available within the article and its Supporting Information.

## Supporting Information Available

Supporting Information is available with the structures optimized in this work and the Cartesian coordinates obtained from the Z-matrix information on ref (30) (ZIP)
Complete data (ZIP)
Employed RI-RPA auxiliary basis set for the lanthanide SMMs (TXT)
